# Deciphering mechanisms of drug sensitivity and resistance to Selective Inhibitor of Nuclear Export (SINE) compounds

**DOI:** 10.1186/s12885-015-1790-z

**Published:** 2015-11-17

**Authors:** Marsha Crochiere, Trinayan Kashyap, Ori Kalid, Sharon Shechter, Boris Klebanov, William Senapedis, Jean-Richard Saint-Martin, Yosef Landesman

**Affiliations:** Karyopharm Therapeutics Inc., 85 Wells Avenue, Newton, MA 02459 USA

**Keywords:** XPO1, Resistance, SINE, Cancer

## Abstract

**Background:**

Exportin 1 (XPO1) is a well-characterized nuclear export protein whose expression is up-regulated in many types of cancers and functions to transport key tumor suppressor proteins (TSPs) from the nucleus. Karyopharm Therapeutics has developed a series of small-molecule Selective Inhibitor of Nuclear Export (SINE) compounds, which have been shown to block XPO1 function both in vitro and in vivo. The drug candidate, selinexor (KPT-330), is currently in Phase-II/IIb clinical trials for treatment of both hematologic and solid tumors. The present study sought to decipher the mechanisms that render cells either sensitive or resistant to treatment with SINE compounds, represented by KPT-185, an early analogue of KPT-330.

**Methods:**

Using the human fibrosarcoma HT1080 cell line, resistance to SINE was acquired over a period of 10 months of constant incubation with increasing concentration of KPT-185. Cell viability was assayed by MTT. Immunofluorescence was used to compare nuclear export of TSPs. Fluorescence activated cell sorting (FACS), quantitative polymerase chain reaction (qPCR), and immunoblots were used to measure effects on cell cycle, gene expression, and cell death. RNA from naïve and drug treated parental and resistant cells was analyzed by Affymetrix microarrays.

**Results:**

Treatment of HT1080 cells with gradually increasing concentrations of SINE resulted in > 100 fold decrease in sensitivity to SINE cytotoxicity. Resistant cells displayed prolonged cell cycle, reduced nuclear accumulation of TSPs, and similar changes in protein expression compared to parental cells, however the magnitude of the protein expression changes were more significant in parental cells. Microarray analyses comparing parental to resistant cells indicate that a number of key signaling pathways were altered in resistant cells including expression changes in genes involved in adhesion, apoptosis, and inflammation. While the patterns of changes in transcription following drug treatment are similar in parental and resistant cells, the extent of response was more robust in the parental cells.

**Conclusions:**

These results suggest that SINE resistance is conferred by alterations in signaling pathways downstream of XPO1 inhibition. Modulation of these pathways could potentially overcome the resistance to nuclear export inhibitors.

**Electronic supplementary material:**

The online version of this article (doi:10.1186/s12885-015-1790-z) contains supplementary material, which is available to authorized users.

## Background

One of the hallmarks of cancer is the inactivation of tumor suppressor proteins (TSPs) resulting from their mislocalization within the cell. Exclusion of TSPs from the nucleus prevents them from activating cell cycle checkpoints, inducing cell cycle arrest, and initiating apoptosis resulting in unrestricted tumor cell propagation. Exportin 1 (XPO1, also known as CRM1) is a member of the karyopherin-β protein family that is responsible for a majority of the nuclear-cytoplasmic protein shuttling [reviewed in [1]]. XPO1 primarily functions as a nuclear export protein whose expression is highly up-regulated in many types of aggressive cancers including glioblastoma [2], ovarian [3], osteosarcoma [4], pancreatic [5], cervical [6], renal [7], metastatic melanoma [8], mantle cell lymphoma [9], acute myeloid leukemia [10], multiple myeloma [11, 12], and leukemia [13] and is the sole transporter of the key TSPs and regulatory proteins p53 [14, 15], p73 [16], p21^CIP^ [17], p27^KIP1^ [18], FOXO [19], IĸB [20], Rb [21], and BRCA1 [22], as well as >200 other cargoes [23]. In conjunction with RanGTP and RanBP3, nuclear XPO1 binds to the leucine-rich nuclear export signal (NES) of a particular cargo protein and transports it through the nuclear pore complex to the cytoplasm. Then RanGTP is hydrolyzed to RanGDP through combined action of RanGAP and RanBP1 resulting in the dissociation of the XPO1/protein complex [reviewed in [24]].

Leptomycin B (LMB) [25] is a well-characterized natural small molecule inhibitor of XPO1 [26] which forms an irreversible covalent bond to Cys528 in the XPO1 NES binding pocket thereby preventing the interaction between XPO1 and its cargo [27]. LMB, however, failed as a therapy due to poor tolerability in the clinic [28]. Subsequently, synthetic inhibitors of XPO1 have been developed including the LMB analog KOS-2464 [17], the maleimide CBS9106 [29], a series of N-azolylacrylates [30], and Karyopharm SINE compounds. SINE compounds covalently bind to Cys528 of XPO1 and appear to be released from the protein in a slowly reversible manner [31–33]. The effect of SINE compounds on a variety of cancer types has been extensively evaluated in preclinical settings, including mantle cell lymphoma [9, 34], non-Hodgkin’s lymphoma [35], multiple myeloma [11, 12], leukemia [32, 36], acute myeloid leukemia [10, 13, 37], chronic lymphocytic leukemia [31, 38], triple-negative breast cancer [39], renal cell carcinoma [7, 40], pancreatic cancer [16, 41], melanoma [42, 43], non-small cell lung cancer [44, 45], glioblastoma [46], hepatocellular carcinoma [47], esophageal squamous cell carcinoma [48], and prostate cancer [49, 50]. The oral drug candidate, selinexor (KPT-330), is currently in both phase 1 and phase 2 clinical trials (Clinicaltrials.gov) for the treatment of hematological as well as solid tumors. Selinexor is well tolerated and shows therapeutic promise (Phase 1 clinical trial manuscripts in preparation).

Although many drugs are initially effective in killing cancer cells, the likelihood for a tumor to develop resistance to a particular drug is a reality that must be anticipated. Many mechanisms exist which may render a cell resistant to drug treatment, both intrinsic and acquired, such as chemical inactivation of the drug, changes in DNA repair mechanisms, delayed apoptosis, increased drug efflux, down-regulation of the drug target or pro-apoptotic factors, changes in drug metabolism, and drug target modifications [reviewed in [51]], as well as alterations in the intracellular localization of a particular protein(s) [17]. In an effort to predict potential mechanisms of resistance that may arise during clinical treatment with SINE compounds, we have established SINE compound-resistant cells from the parental SINE compound-sensitive HT1080 fibrosarcoma (*wt* p53) cell line [52]. The response of resistant and parental cells to treatment with SINE compounds was compared by examining changes in proliferation, cell cycle phases, protein localization and expression, and gene expression profiles. In addition, the DNA sequence of the XPO1 cargo-binding pocket, the ability of XPO1 to bind drug, as well as drug efflux activity was evaluated in parental and resistant cells. The findings presented in this study indicate that developing resistance to SINE compounds is a prolonged process that involves modulating the expression of genes downstream of XPO1 inhibition that are involved in pathways such as inflammation, cell adhesion, and apoptosis, and provide guidance for future studies to test the inhibition of these pathways in combination with selinexor in order to overcome resistance.

## Methods

### Cell culture and reagents

HT1080 cell lines (ATCC) were cultured in EMEM, Neo-NHEK (Lonza) was cultured in KGM-Gold, HaCAT (AddexBio) was cultured in DMEM, and leukocytes were isolated from healthy donor whole blood by the Buffer EL (Erythrocyte Lysis Buffer, Qiagen) method and cultured ex vivo in RPMI. Media were supplemented with 10 % heat-inactivated fetal bovine serum (FBS, Gibco), 100 units/mL penicillin, 100 μg/mL streptomycin (Gibco), and 1× GlutaMAX (Gibco), and maintained in a humidified incubator at 37 °C in 5 % CO_2_. Resistant HT1080 cells were initiated in the presence of 5 nM KPT-185 and over the course of approximately 10 months the concentration was gradually escalated to 600 nM. The XPO1 SINE compounds KPT-185, KPT-251, and KPT-330 were synthesized at Karyopharm Therapeutics, Inc. (Newton, MA).

### Clonogenic survival assay

HT1080 parental and resistant cells were plated at 5000 cells/well in 12 well plates (Cell Treat). The following day cells were treated with either DMSO (Sigma) or with KPT-185 (0, 3.7, 12.3, 111, 333, or 1000 nM for generation of resistance, or 1 μM to evaluate resistance). On days 0, 4, 6, and 8 cells were fixed and stained with Gentian Violet (RICCA Chemical Company) and imaged with a digital camera (Sony Cybershot).

### MTT assay

Cells from log phase cultures were seeded in 96-well flat-bottom culture plates. Escalating concentrations of KPT-185, KPT-330, KPT-251, or leptomycin B (LMB) were added to the wells and incubated at 37 °C in a 5 % humidified CO_2_ incubator for 72 hours (in triplicate). The CellTiter-Fluor Cell Viability Assay (Promega) was performed as instructed by the manufacturer. The whole procedure was repeated three times. The inhibitory rate of cell growth was calculated using the formula: % Growth inhibition = (1− OD extract treated)/OD negative control × 100) [53].

### Flow Cytometry

Cell cycle profile analysis was performed using the BrdU Flow Kit (BD Pharmingen) according to the manufacture’s protocol. Briefly, HT1080 parental and resistant cells were plated in 6 well plates at 500,000 cells/well. Cells were treated with either DMSO or 600 nM KPT-185. Prior to harvesting, HT1080 parental cells were incubated with 10 μM BrdU for 2 hours while HT1080 resistant cells were incubated with 10 μM BrdU for 4 hours. Cells were fixed and stained for BrdU and 7-AAD according to the manufacturer’s protocol. Cells were then analyzed on a BD LSRFortessa (BD Biosciences) at the Dana Farber Cancer Institute (Boston, MA) and the data was subsequently analyzed using FCS Express 4 software (De Novo Software).

### Immunofluorescence

HT1080 parental and resistant cells were plated on glass coverslips (BioCoat, BD Biosciences) at 500,000 cells/well in 6 well plates and grown overnight. Cells were treated with 1 μM KPT −185 for either 4 hours to detect p53 and IkB or for 24 hours to detect p21, p27, FOXO-1, and PP2A. After treatment, coverslips were washed with 1× PBS (phosphate buffered saline) then fixed in either 3 % paraformaldehyde buffer (3 % paraformaldehyde/2 % sucrose/1× PBS) or 100 % ice-cold methanol for 15 min then washed with 1× PBS. Cells were permeabilized with 0.1 % Triton X-100/1 % BSA/1× PBS (PFA fixation) or 0.1 % Tween 20/0.3 M glycine/1 % BSA/1× PBS (Methanol fixation) for at least 30 minutes. After washing 3 times with 1× PBS, cells were stained overnight with the corresponding antibodies listed above diluted in 1%BSA/1× PBS. Protein signal was detected with species specific Alexa Fluor 488 secondary antibodies (Invitrogen) while DNA was stained with DAPI (Invitrogen). Protein localization was visualized with a Nikon Eclipse Ti inverted fluorescence microscope (Nikon) and monochrome camera (ANDOR).

### Western blot

HT1080 parental and resistant cells were plated at 375,000 cells/well in 6 well plates and treated with either DMSO (0) or 0.03, 0.1, 0.6, 1, or 3 μM KPT-185 for 24 hours prior to collection by trypsinization. Proteins were extracted from cells in Pierce RIPA buffer (Thermo Scientific) supplemented with phosphatase and protease inhibitors (Roche), quantified by the Pierce BCA Protein Assay Kit (Thermo Scientific), and normalized such that for each sample 10 μg of total protein was loaded per lane. Proteins were separated by loading on Novex NuPAGE 4–12 % Bis-Tris Gels (Life Technologies) and transferring to nitrocellulose with the Novex iBlot Gel Transfer Stacks (Life Technologies). The following primary antibodies were used for immunoblot analysis: XPO1 (Santa Cruz), p53 (Santa Cruz), p21 (Abcam), PARP (Cell Signaling), Caspase 3 (Abcam), cleaved Caspase 3 (Cell Signaling), Mcl-1 (Santa Cruz), p-pRb (Cell Signaling), pRb (Cell Signaling), and β-actin (Santa Cruz). Protein signals were detected with infrared linked species-specific secondary antibodies (LI-COR Biosciences). Western blot images were detected with the ODYSSEY Infrared Imaging System (LI-COR Biosciences).

### Microarray analysis

HT1080 parental and resistant cells were grown for 8 hours in either control DMSO (untreated) or 600 nM KPT-185 (treated) and each condition was prepared in triplicate. Cells were harvested and total RNA was extracted from the 12 independent preparations (3 repetitions of each sample) from untreated parental, untreated resistant, treated parental, and treated resistant. The RNA was submitted to Asuragen and was then quality assured and reverse transcribed to cDNA. Microarray data was collected at Asuragen using GeneChip Affymetrix HuGene10stv1_Hs_ENTREZG_desc array, according to standardized operating procedures. Microarray data was then interrogated with the MetaCore software suite from Thomson Reuters.

### Quantitative real-time PCR

Cells were cultured with vehicle or KPT-185 for 4 or 24 hours, then cells were collected and total RNA was purified using the QIAmp RNA Blood Mini Kit (Qiagen), including treatment with DNAse (Qiagen). cDNA was reversed transcribed from the purified RNA using the High Capacity cDNA Reverse Transcriptase Kit (Life Technologies). Quantitative real-time PCR was performed with Taqman probes for a subset of genes (see Table 7) and GAPDH (Life Technologies) using the ViiA 7 Real-Time PCR System (Life Technologies).

## Results

**Fig. 1:**
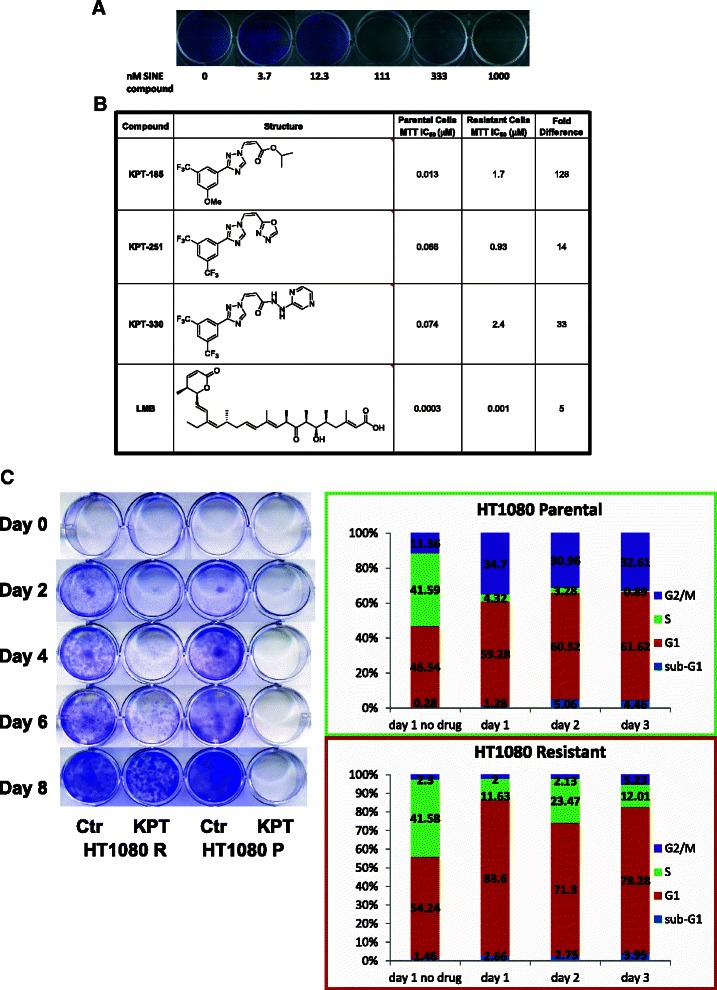
Generation of SINE compound-resistant HT1080 cells. **a** HT1080 parental cells grown in 0, 3.7, 12.3, 111, 333, and 1000 nM KPT-185 in an 8 day clonal assay (stained with gentian violet) for the selection of the KPT concentration to initiate generation of the resistant cell line. **b** IC_50_ values for parental and resistant cells treated with KPT-185, KPT-330, and LMB, determined in an MTT assay. **c** HT1080 parental and resistant cells grown in 1 μM KPT-185 in the 8 day clonal growth assay (stained with gentian violet). **d** FACS cell-cycle analysis of HT1080 parental and resistant cells treated with 0 or 600 nM KPT-185 for 1–3 days

Resistance to drugs by cancer cells is a major obstacle in cancer therapy. In an effort to evaluate the ability of a cancer cell to overcome SINE compound-mediated cancer cell death, we sought to create a SINE compound-resistant cancer cell line (Fig. 1). Due to its wt p53 status, ease of cultivation, and sensitivity to SINE compounds, the HT1080 fibrosarcoma cell line was chosen for this endeavor. In order to determine the starting concentration of SINE compound to generate the resistant cells, HT1080 cells were initially grown in increasing concentrations of the SINE compound KPT-185 for 8 days and evaluated for growth by the clonal growth assay (Fig. 1a). Cells were able to grow in KPT-185 at concentrations up to 12.3 nM but not at 111 nM (evaluated by gentian violet staining). Consequently, 5 nM KPT-185 was selected as the initial concentration for the selection of the resistant cells. Over the course of approximately 10 months, the cells were cultured in the presence of gradually escalating concentrations of KPT-185 until the concentration reached 600 nM. It was at this point that the cells were considered “resistant” and evaluated for their response to KPT-185 treatment compared to sensitive, parental HT1080 cells.

We then compared the effects of SINE compounds on the viability of resistant versus parental cells (Fig. 1b). An approximately 130-fold reduction in sensitivity was observed for KPT-185 (IC_50_ of 0.013 μM in parental cells compared with an IC_50_ of 1.7 μM in resistant cells). Next we determined whether resistance was specific for KPT-185 or would also be observed with two additional, structurally related SINE compounds, KPT-330 and KPT-251. An approximately 33-fold reduction was measured for KPT-330 (0.074 μM compared to 2.4 μM in parental and resistant cells, respectively) and for KPT-251 an ~14-fold difference in sensitivity was observed (IC_50_ of 0.066 μM compared to 0.93 μM in parental and resistant cells, respectively). This data suggests that the KPT-185 resistant cells are also resistant to additional analogues of this class of SINE compounds. Resistant cells were also slightly less sensitive to LMB, with an IC_50_ of 0.0014 μM compared to 0.0003 μM for parental cells.

Next, we sought to characterize the differences in response to treatment with SINE compounds between parental and resistant cells. We first tested the cells by the clonal growth assay. Parental and resistant cells were incubated with 1 μM of KPT-185 for 8 days and then evaluated for survival and cell division (Fig. 1c). Parental cells did not survive in KPT-185 whereas resistant cells were able to proliferate in culture in the presence of 1 μM KPT-185. Next, we tested by Fluorescence Activated Cell Sorting (FACS) the effects of KPT-185 on the cell cycle (Fig. 1d). Cells were incubated with or without 0.6 μM KPT-185 and their cell cycle distribution profile was evaluated daily. The results show that parental and resistant cells have different cell cycle profiles when incubated with KPT-185. Parental cells show a dramatic (≥10-fold) reduction in S-phase and an approximately 3-fold increase in G2/M phases while resistant cells show a less marked reduction in S-phase (2- to 4-fold) with little change in G2/M. Taken together, these data demonstrate that resistant cells, unlike parental cells, are much less likely to undergo cell cycle arrest at G2/M and are able to survive and continue to proliferate, albeit more slowly, in the presence of KPT-185.

**Fig. 2:**
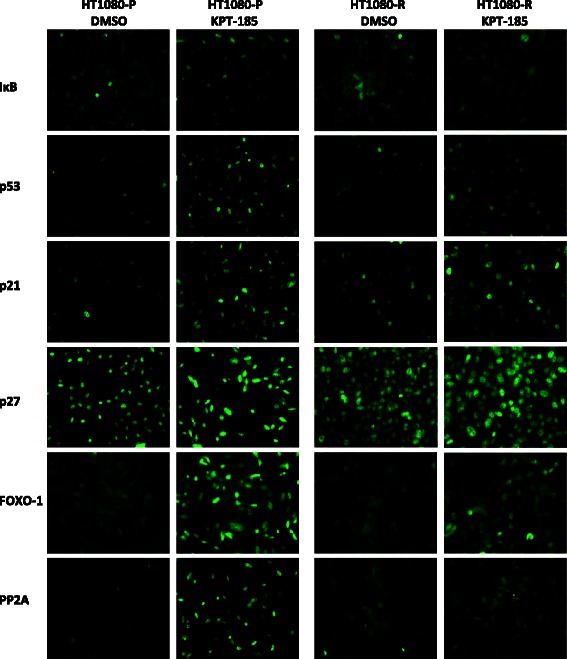
SINE compound-induced nuclear localization is impaired in resistant HT1080 cells. Parental and resistant cells were treated with either DMSO or 1 μM KPT-185 and then evaluated by immunofluorescence for the subcellular localization of IkB, p53, p21, p27, FOXO-1, and PP2A

Previous studies have shown that XPO1 inhibition with SINE compounds induces nuclear accumulation of its cargoes, which include most of the major tumor suppressor and cell cycle regulatory proteins [reviewed in [54–56]]. The ability of SINE compounds to force nuclear retention of XPO1 cargoes was compared in parental and resistant cells (Fig. 2). Both cell lines were treated with either control (DMSO) or 1 μM KPT-185 and evaluated by immunofluorescent analysis for the subcellular localization of IkB and p53 after 4 hours of treatment, and for p21, p27, FOXO-1, and PP2A after 24 hours of treatment. In parental cells, KPT-185 forced effective nuclear localization of all six cargoes examined. However, in the resistant cells treated with KPT-185, the nuclear localization of the six cargoes was less intense than in parental cells, with certain cargoes retaining cytoplasmic localization, particularly for IkB, p27, FOXO-1, and PP2A. These data indicate that SINE compounds are less effective at inducing nuclear accumulation of XPO1 cargoes in resistant cells.

**Fig. 3:**
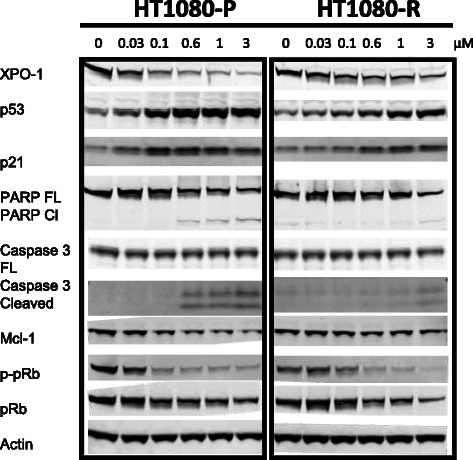
SINE compound-mediated effects on protein expression in HT1080 parental versus resistant cells show differential dose–response. Parental and resistant cells were treated with 0, 0.03, 0.1, 0.6, 1, and 3 μM KPT-185 for 24 hours and then evaluated by immunoblot analysis for the expression of XPO1, p53, p21, full-length (FL) and cleaved (Cl) PARP, full-length and cleaved Caspase 3, Mcl-1, p-pRb, pRb, and actin

In addition to increasing nuclear retention of XPO1 cargoes, SINE compounds also modulate the expression levels of several key regulatory proteins [9, 10, 37, 42]. In order to compare the steady state protein expression profiles of regulatory proteins, both parental and resistant cells were treated with increasing concentrations of KPT-185 up to 3 μM for 24 hours followed by immunoblot analysis (Fig. 3). For all proteins evaluated, the observed expression changes occurred at lower compound concentrations in parental cells compared to resistant cells. The levels of XPO1 protein was substantially decreased in parental cells treated with 0.03 μM KPT-185 and decreased dramatically at 0.1 μM whereas in resistant cells a comparable level of reduced expression was not reached until treatment with 3 μM KPT-185. Induction of the expression of the TSPs p53 and p21 also occurred at lower KPT-185 concentrations in parental than resistant cells. Induction of apoptosis, as indicated by cleavage of PARP and Caspase 3, was observed at 0.6 μM KPT-185 in parental cells but was not detected in resistant cells until treatment with 3 μM KPT-185. In line with the induction of apoptosis with SINE compound treatment, a greater decrease in the expression of the pro-survival protein Mcl-1 was detected in parental cells compared to resistant cells. Likewise, dephosphorylation of the inactive pRb form, in response to KPT-185, occurred at lower KPT-185 concentrations in parental cells compared to resistant cells. Taken together, SINE compound-mediated changes in the levels of key regulatory proteins indicate that cell cycle arrest and apoptosis are initiated at lower drug concentrations in parental cells than in resistant cells.

Recently, Neggers and colleagues reported that the sensitivity of T-cell lymphoma to SINE compounds was reduced by more than 250-fold by a knock-in experiment when cysteine 528, the residue that binds SINE compounds, as well as LMB, was mutated to serine on XPO1 [57]. To determine whether resistance in HT1080 was the result of mutations in cysteine 528, DNA from resistant and parental cells was extracted and the XPO1 gene was sequenced. The sequence of the XPO1 cargo-binding pocket was identical between parental and resistant cells (data not shown). Therefore, resistance of HT1080 cells to KPT-185 is not associated with mutations in the SINE compounds binding site of XPO1.

To test whether there was an alteration in drug-target interaction in resistant cells, both parental and resistant cells were tested in an XPO1 occupancy assay (manuscript in preparation). In this assay, cells were treated with serially increasing amounts of SINE compound followed by the addition of biotinylated LMB and then evaluated for the amount of XPO1 bound to drug compared to that bound to biotinylated LMB. The results from this assay showed that resistant and parental cells had the same XPO1 occupancy values (data not shown). This indicates that reduction of the drug-target interaction was not the means of conferring resistance in the HT1080 cells.

Next, we sought to determine whether resistance was the result of the activation of common multidrug resistance mechanisms. Parental and resistant cells were incubated with Cyclosporin A or Verapamil, which are the competitive and non-competitive inhibitors of P-Glycoprotein, the multidrug resistance MDR1 gene product, and the multidrug resistance associated proteins (MRP). Both proteins are members of the superfamily of ATP-binding cassette (ABC) transporters. Calcein A was used as a substrate to quantify the transporter activity. The analyses revealed similar accumulation of calcein A in untreated and treated parental and resistant cells (not shown). This indicated that resistant cells did not gain activation of these multidrug resistance transporters. Taken together, the above results suggest that development of resistance to SINE compounds was not the result of mutations in the cargo binding pocket of XPO1 or other modification that reduced drug-target binding, or activation of a common multidrug resistance mechanism that exclude drug from the cell cytoplasm. Therefore, resistance is likely achieved through modulation of an inhibitory pathway(s) downstream of XPO1 inhibition.

In an effort to identify SINE compound resistance mechanisms exploited by HT1080 cells, we used gene expression profiling analysis to compare expression patterns between parental and resistant cells. Previous observations showed that XPO1 inhibition can be detected as early as 30 minutes following SINE compound treatment with maximal cargo retention in the nucleus occurring between 4 and 8 hours following treatment. To allow for adequate nuclear accumulation of XPO1 cargoes and the initiation of gene transcription we chose to harvest the cells for analysis following 8 hours of drug treatment.

In order to identify mechanisms associated with resistance to KPT-185, the gene expression profiles between resistant and parental cells were compared at baseline (prior to drug treatment). In addition, the mechanism of drug action and how it changes when resistance is obtained was revealed when we also compared the differential transcriptional response of parental and resistant cells to drug treatment.

Using a fold shift cut-off of +/− 1.5 fold change, analysis of the microarray data with the Metacore program identified the following functional gene groups to be the most differentially regulated between untreated resistant and untreated parental cells: cell adhesion (Table 1, where red = increased in resistant, blue = decreased in resistant, color intensity corresponds to fold change magnitude), apoptosis (Table 2), and inflammation (Table 3). The cell adhesion group had the highest number of genes that were differentially expressed between untreated resistant and parental cells (80 genes, Table 1), followed by inflammation (63 genes, Table 3), and then apoptosis (37 genes, Table 2). Although three functional groups were identified, the expression patterns of several genes were found to overlap in these groups. For example, chemokine (C-C motif) ligand 2 (CCL2) was the most differentially expressed gene in resistant versus parental cells (13.92 fold) and was identified by the Metacore program to be associated with both cell adhesion and inflammation pathways, while triggering receptor expressed on myeloid cells 1 (TREM1) had the second highest level of differential expression in resistant versus parental cells (6.89 fold) and was identified in both apoptosis and inflammation related pathways. Integrin, alpha 11 (ITGA11) was the most down-regulated gene in resistant versus parental cells (−4.12 fold) and was identified in both cell adhesion and apoptosis related pathways, while phospholipase A2, group IVA (cytosolic, calcium-dependent) (PLAG42A) was one of the most down-regulated genes in resistant versus parental cells (−3.67 fold) and was identified in all three pathways. The modulation of the expression of these genes known to be involved in these three functional groups reinforces the likelihood that resistance is not conferred by alterations in one single pathway but instead by the combined effect of multiple pathways.

**Table 1:**
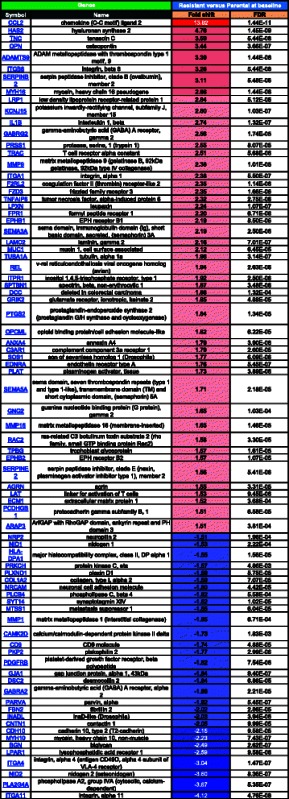
MetaCore analysis of fold changes in expression of cell adhesion-related genes in SINE compound-resistant versus parental cells pre-treatment

FDR = Benjamini-Hochberg False Discovery Rate. Red = positive, blue = negative, color intensity corresponds to fold change magnitude

**Table 2:**
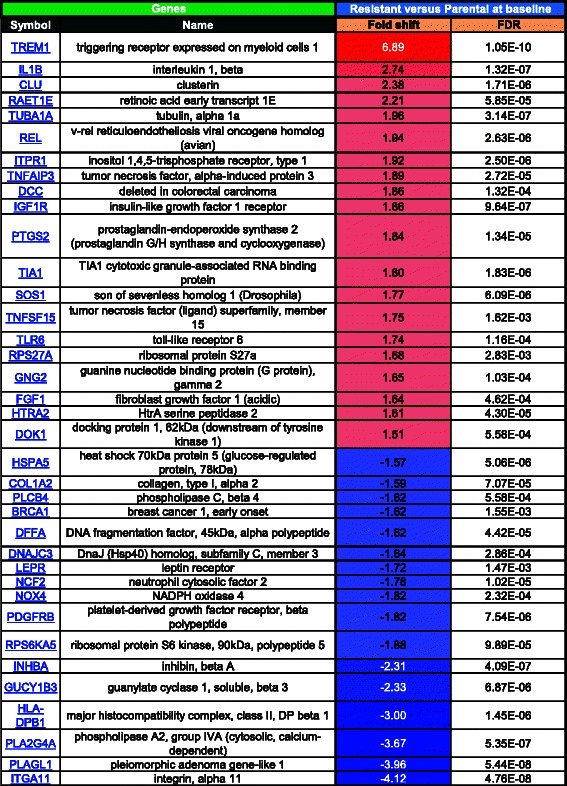
MetaCore analysis of fold changes in expression of apoptosis-related genes in SINE compound-resistant versus parental cells pretreatment

FDR = Benjamini-Hochberg False Discovery Rate. Red = positive, blue = negative, color intensity corresponds to fold change magnitude

**Table 3:**
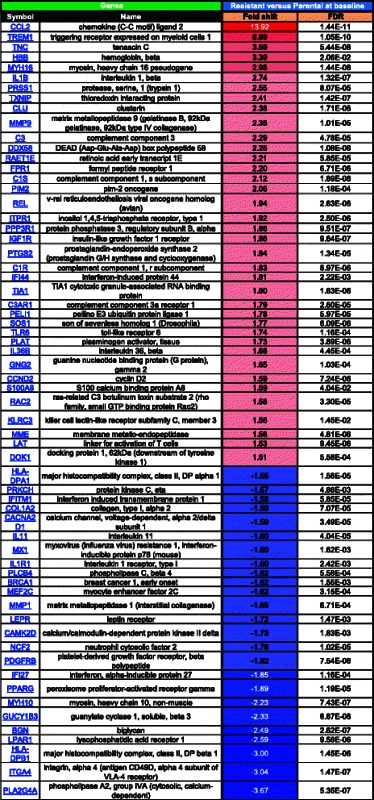
MetaCore analysis of fold changes in inflammation-related genes in SINE compound-resistant versus parental cells pre-treatment

FDR = Benjamini-Hochberg False Discovery Rate. Red = positive, blue = negative, color intensity corresponds to fold change magnitude

**Fig. 4:**
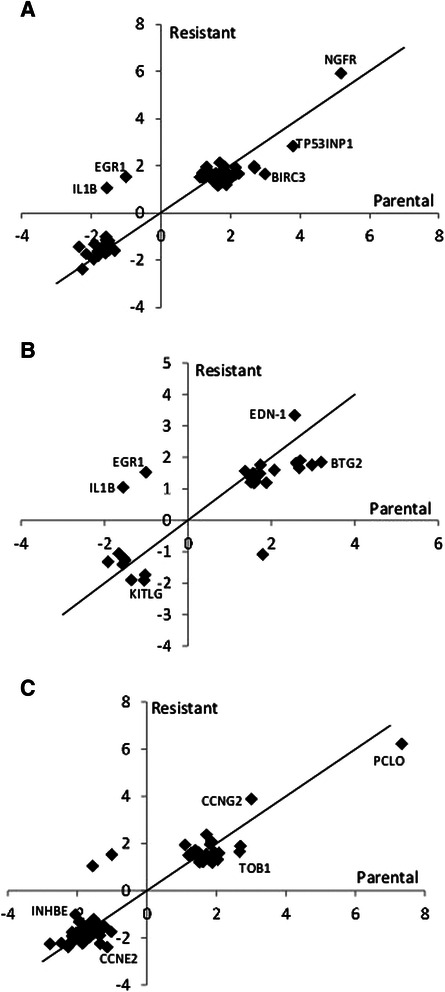
Microarray quadrant analysis for differentially expressed genes in parental versus resistant SINE compound-treated cells. Untreated parental cells were compared to treated parental cells (x-axis) while untreated resistant cells were compared to treated resistant cells (y-axis) and genes that were 1.5 fold up- and down- regulated in each of the SINE compound treated cell lines were plotted. **a** Fold change in mRNA expression following KPT-185 treatment of apoptosis and autophagy-related genes. NGFR induction was similar in both cell lines, while TP53INP1 and BIRC3 induction was stronger in parental cells; EGR-1 was induced exclusively in resistant cells while IL1B was down-regulated only in parental cells. **b** Fold change in mRNA expression following KPT-185 treatment of proliferation-related genes. EDN-1 induction was stronger in resistant cells; BTG2 induction was stronger in parental cells; KITLG was exclusively down-regulated in resistant cells. **c** Fold change in mRNA expression following KPT-185 treatment of cell-cycle and cytoskeleton-related genes. PCLO induction was stronger in parental cells; CCNG2 induction was stronger in resistant cells; TOB1 induction was stronger in parental cells; CCNE2 and INHBE were exclusively down-regulated in resistant and parental cells, respectively. Black line represents perfect correlation as reference

We next sought to investigate the differential gene expression in response to drug treatment in parental sensitive compared to resistant cells. For this analysis, expression patterns in the parental and resistant cells at baseline (untreated) were compared to their respective SINE compound treated counterparts to generate lists of genes that are at least 1.5 fold up- and down-regulated in each cell line in response to SINE compound treatment. Pathway analysis comparing these lists found that the majority of gene transcription changes for both comparisons were part of one of three main categories, 1) apoptosis and autophagy (Table 4, Fig. 4a), 2) proliferation (Table 5, Fig. 4b), and 3) cell cycle and cytoskeleton (Table 6, Fig. 4c). Comparison of the response to drug treatment in parental and resistant cells for genes with changes of 1.5 fold or greater showed that, in general, for both the apoptosis and autophagy and proliferation categories, more genes were up-regulated in response to drug treatment in both parental and resistant cells than down-regulated, whereas in the cell cycle and cytoskeleton category more genes were down-regulated in response to drug treatment in both parental and resistant cells.

**Table 4:**
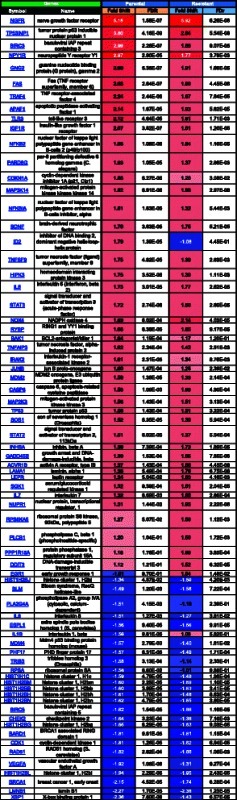
MetaCore analysis of fold changes in expression of apoptosis and autophagy-related genes in SINE compound-resistant versus parental cells post-treatment

FDR = Benjamini-Hochberg False Discovery Rate. Red = positive, blue = negative, color intensity corresponds to fold change magnitude

**Table 5:**
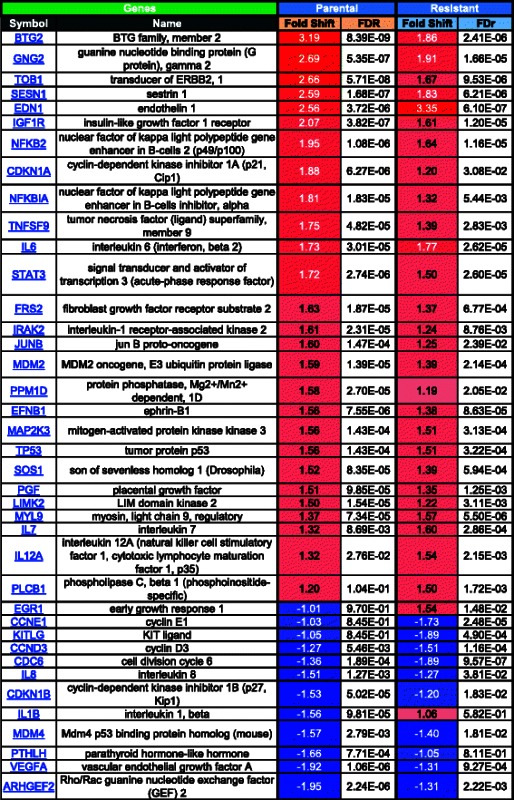
MetaCore analysis of fold changes in expression of proliferation-related genes in SINE compound-resistant versus parental cells post-treatment

FDR = Benjamini-Hochberg False Discovery Rate. Red = positive, blue = negative, color intensity corresponds to fold change magnitude

**Table 6:**
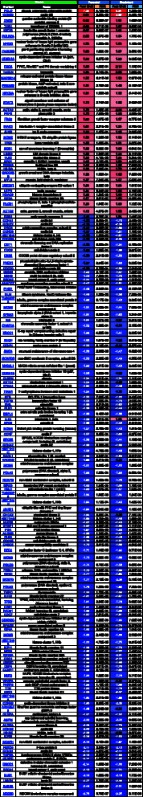
MetaCore analysis of fold changes in expression of cell cycle and cytoskeleton-related genes in SINE compound-resistant versus parental cells post-treatment

FDR = Benjamini-Hochberg False Discovery Rate. Red = positive, blue = negative, color intensity corresponds to fold change magnitude

In the apoptosis and autophagy category, treated parental cells had more differentially expressed genes (≥1.5-fold change) than treated resistant cells, with 33 genes up-regulated in parental cells compared to 31 genes resistant cells, and 24 genes down-regulated in parental cells compared to 14 genes resistant cells (Table 4). In the proliferation category, parental cells again had more differentially expressed genes than resistant cells in response to drug treatment, with 23 genes up-regulated in parental cells compared to 16 genes in resistant cells, and 7 genes down-regulated in parental cells compared to 4 genes in resistant cells (Table 5). Lastly, in the cell cycle and cytoskeleton category, 25 genes were up-regulated in parental cells compared to 26 genes in resistant cells, while 97 genes were down-regulated in parental cells compared to 83 genes in resistant cells (Table 6).

Overall, drug treatment induced changes in gene expression in the same direction in both parental and resistant cells with a few exceptions. In the apoptosis and autophagy category, inhibitor of DNA binding 2, dominant negative helix-loop-helix protein (ID2) was up-regulated in parental cells (1.79 fold) with no change in resistant cells (−1.08 fold), whereas in all 3 categories early growth response 1 (EGR1) was up-regulated in resistant cells (1.54 fold) with no change in parental cells (−1.01 fold), and interleukin 1, beta (IL1B) was down-regulated in parental cells (−1.56 fold) with no change in resistant cells (1.06 fold). These results suggest that parental cells had a more robust transcriptional response to drug treatment than resistant cells.

Next, microarray quadrant analysis was used to plot the expression changes in response to drug treatment between parental and resistant cells for each category (Fig. 4). Fold-change values for genes found to be at least 1.5 fold up- or down-regulated in resistant cells were plotted on the y-axis while fold-change values for genes found to be at least 1.5 fold up- or down-regulated in parental cells were plotted on the x-axis. In this visualization, any genes falling on a diagonal line with a slope of 1 and intercept of zero demonstrate perfect correlation between the two cell lines whereas genes that fall significantly above or below the diagonal show differential response between resistant and parental cells. Most of the genes clustered along the diagonal in the top right and bottom left quadrants of the apoptosis and autophagy (Fig. 4a), proliferation (Fig. 4b), and cell cycle and cytoskeleton (Fig. 4c) categories demonstrating the tendency for the genes in these categories to follow the same trend in expression in response to drug treatment in both parental and resistant cells. A few genes, however, did not fall along the diagonal indicating their expression to be differentially affected by drug treatment in one cell type compared to the other. For example, in the apoptosis and autophagy category, nerve growth factor receptor (NGFR) induction was similar in both parental and resistant cells, while tumor protein p53 inducible nuclear protein 1 (TP53INP1) and baculoviral IAP repeat containing 3 (BIRC3) were both more highly expressed in parental compared to resistant cells (Fig. 4a). In the proliferation category, BTG family, member 2 (BTG2) was more highly expressed in parental cells, endothelin 1 (END1) was more highly expressed in resistant cells, and KIT ligand (KITLG) was down-regulated in resistant cells (Fig. 4b). Lastly, in the cell cycle and cytoskeleton category, piccolo presynaptic cytomatrix protein (PCLO) expression was higher in parental cells, cyclin G2 (CCNG2) expression was higher in resistant cells, and transducer of ERBB2, 1 (TOB1) expression was higher in parental cells, while cyclin E2 (CCNE2) was down-regulated in resistant cells and inhibin, beta E (INHBE) was down-regulated in parental cells. These data indicate that regardless of sensitivity, most genes, with a few exceptions, respond with similar patterns in expression in the presence of drug.

The full list of genes showing at least 1.5 fold change in expression following drug treatment in either parental or resistant cells can be found in Additional file 1: Table S1. Interestingly, of these 894 genes (out of 13,951 total genes), none of the genes had fold changes that were expressed +/− 1.5 fold in opposing directions in parental compared resistant cells (Additional file 1: Table S1).

Together, these results suggest that inhibition of XPO1 affects multiple downstream pathways involving hundreds of genes. Moreover, drug exposure resulted in similar patterns of gene expression changes in both parental and resistant cells. However, the extent of the response in parental cells was much stronger than that in the resistant cells.

In an effort to validate the microarray data, a subset of genes that were found to be up-regulated in both parental and resistant cells in response to drug treatment were tested by real-time quantitative PCR (qPCR). For this validation, HT1080 parental and resistant cells, as well as primary normal keratinocytes (Neo-NHEK) and the keratinocyte cell line HaCaT were tested in vitro, while normal human leukocytes were isolated from donor blood and tested ex vivo. Genes were selected for validation based on an arbitrary fold change cutoff of 2.5 and those genes containing regulatory elements that are activated by TSPs such as p53 and FOXO were identified (Table 7). qPCR analysis showed that all of the 19 genes selected from the microarray data were up-regulated in both parental and resistant HT1080 cells in response to drug treatment, thus confirming the microarray results. In both the parental and resistant HT1080 cells types, genes were induced between 2- and 400-fold. Genes such as solute carrier family 16, member 6 (SLC16A6), solute carrier family 43 (amino acid system L transporter) member 2 (SLC43A2), arrestin domain containing 3 (ARRDC3), nerve growth factor receptor (NGFR), and heat shock 70 kDa protein 4-like (HSPA4L) were highly up-regulated in all cell types in response to treatment with SINE compounds, exemplifying the effect of inhibiting XPO1 protein in both malignant and normal cells. Many of these validated genes also contain XPO1 cargo transcription factor binding elements, supporting the observation that inhibition of XPO1 by SINE compounds forces nuclear retention of TSPs allowing them to be functionally active in the nucleus and drive transcription of their target genes.

**Table 7:** RT-PCR verification of microarray gene expression and identifcation of XPO1 cargoes containing a TSP 5’ regulatory element(s)

Genes	HT1080 Parental Fold Induction Microarray	HT1080 Resistant Fold Induction Microarray	HT1080	HT080	Neo-NHEK	HaCaT	Leukocytes Fold Induction RT-PCR	Presence of XPO-1 cargo transcription factor binding element
			Parental Fold Induction RT-PCR	Resistant Fold Induction RT-PCR	Fold Induction RT-PCR	Fold Induction RT-PCR		
SLC16A6	4.04	7.05	10X	14X	3X (24hrs)	10X (4hrs)	30X (4hrs)	FOXO1
SLC16A9	4.45	7.7	4X	16X	3.5X (4hrs)	No induction	No expression	NF-AT
BIRC3	2.99	1.65	3X	2.5X	3X (24hrs)	2X (24hrs)	No Induction	p53
SLC43A2	6.41	6.99	12X	16X	6X	4X	4.5X (4hrs)	FOXO1
GNG2	2.69	1.91	3X	2.5X	4X (4hrs)	20X	No Induction	
ACER2	2.71	2.01	4X	2.5X	3.5X (24hrs)	No Induction	2.5X (24hrs)	p53
BTG2	3.19	1.86	3.5X	2X	2.5X (24hrs)	No Induction	No Induction	p53, FOXO1
RRAGD	2.76	2.55	3X	3X	2 X	No expression	7X	AP-1
NPY1R	2.97	1.77	4X	3.5X	3X (4hrs)	No Induction	3X (24hrs)	p53, NF-AT
SLC44A2	5.33	3.97	8X	9X	2X	No Induction	No Induction	
PLCD4	3.03	3.43	13.5X	9X	2.5X (4hrs)	No Induction	No expression	p53, FOXO1, AP-1
FAS	2.68	1.99	2.5X	2.5X	No Induction	No Induction	No Induction	p53, AP-1
ARRDC3	4.05	4.3	5.5X	7X	8X (24hrs)	5X (4hrs)	5X	FOXO1,3
NGFR	5.18	5.92	400X	70X	43X (4hrs)	5.5X (4hrs)	100X (4hrs)	p53, FOXO4, NF-AT
Tp53INP	3.8	2.84	6X	3X	5X (24hrs)	5.5X (24hrs)	No Induction	
PCLO	7.33	6.24	30X	25X	9X	6X (4hrs)	No Induction	FOXO1,3, NF-AT, AP-1
HSPA4L	4.38	3.32	7X	5X	2X (24hrs)	2X (4hrs)	27X (24hrs)	AP-1
STK32A	8.92	8.4	90X	10X	No expression	No expression	No Induction	
RNF150	9.76	7.9	45X	10X	50X (4hrs)	No Induction	160X (4hrs)	FOXO1

Lastly, because p53 is a major tumor suppressor protein whose function relies on its nuclear retention in response to SINE compound treatment, we sought to interrogate the microarray data for those genes whose expression is known to be regulated (either positively or negatively) by p53. Additional file 1: Table S2 lists all of the differentially expressed genes present in Additional file 1: Table S1 that have p53 regulatory elements (as determined by MetaCore analysis, where “+” is positive, “-“is negative, and “?” is uncertain regulation by p53) from resistant versus parental cells post-treatment.

Together, these results demonstrate that resistant HT1080 cells are not absolutely agnostic to SINE compounds but rather have reduced sensitivity, and that when resistance is conferred multiple pathways are altered thereby providing suggestions for specific pathways that can be targeted for future combination studies with selinexor treatment.

## Discussion

Many cancers develop resistance to treatment, rendering the therapy ineffective and resulting in the onset of a refractory disease. Although resistance to selinexor in the clinic has not been reported, we sought to predict the characteristics of potential SINE resistance mechanisms by creating a SINE compound-resistant cell line from parental fibrosarcoma cells that are sensitive to SINE compound treatment. To identify methods for overcoming resistance, resistant and parental sensitive cells were compared pre-treatment and post-treatment to identify mechanisms leading to the resistant phenotype as well as investigate their differential response to SINE compound treatment.

The extensive period of time required to achieve resistance speaks to the fact that SINE compounds are an effective, robust therapy for killing cancer cells. Development of resistance to the SINE compound KPT-185 required 10 months of continuous exposure in vitro. In comparison, it took 3 months to develop resistance to the tyrokinase inhibitor STI571 by chronic myelogenous leukemia cell lines [58], 6 months to develop resistance to taxol in the human ovarian cancer cell line A2780 [59], and 12 weeks to develop resistance to the HDAC inhibitor valproic acid by renal cell carcinoma Caki-1 cells [60]. Although resistant cells were selected by treatment with KPT-185, these cells were also resistant to KPT-330 (selinexor) as well as to LMB, indicating conservation of the mechanism(s) of resistance across different inhibitors of XPO1.

A characteristic feature of SINE compound treatment on cells both in vitro and in vivo is the nuclear retention of key XPO1 cargoes [reviewed in [54]]. Although certain cargoes are detected in the nuclei of SINE compound-resistant cells treated with KPT-185, nuclear accumulation was greatly reduced compared to parental cells. It is likely that nuclear retention of XPO1 cargoes would be enhanced if resistant cells were treated with higher concentrations of KPT-185 because of the changes observed in the levels of proteins by immunoblot (Fig. 3). In agreement with previously published studies, the XPO1 inhibition in parental cells lead to increased protein levels of p53 [9, 12, 42, 44, 45, 50], which corresponded with increased gene expression of tumor protein p53 inducible nuclear protein 1 (TP53INP) by both parental and resistant cells in the microarray (see Additional file 1: Table S1). Increase in the protein level of the p53 transcriptional target p21 [13, 40, 48] also corresponded with increased gene expression of cyclin-dependent kinase inhibitor 1A (p21, Cip1) (CDKN1A) by parental cells in the microarray (see Additional file 1: Table S1), whereas a decrease in p-pRb [42] and Mcl-1 [12, 31, 46] proteins were observed in both cell types. That the expression of the above proteins was only affected to the same extent in resistant compared to parental cells when the resistant cells were treated with 3 times more drug than parental cells (see Fig. 3) suggests that resistant cells are not strictly “resistant” to SINE compounds but rather are less sensitive.

Evaluation of the effects of SINE compound treatment on the cell cycle as determined by FACS analysis showed both similarities as well as a distinct difference between parental and resistant cells. SINE compound treatment induced G1 arrest with a concomitant decrease in S phase in both parental and resistant cells, which has also been reported in the AML cell lines MV4-11, OCI-AML3, and MOLM-13 [37], and in the kidney cancer cell lines ACHN and 786-O [7]. However, SINE compound treatment only led to arrest in the G2/M phase of parental cells while having no effect on the G2/M phase of resistant cells. This suggests that parental cells arrest in G1 and those cells that were in S phase accumulate in G2/M, whereas resistant cells that exit S phase are able to cycle through G2/M and accumulate in G1, resulting in the higher percentage of resistant cells in G1 at each day post treatment compared to parental cells. The observation that S phase is not completely lost in resistant cells post SINE treatment is indicative of their ability to continue to proliferate in the presence of drug as was observed in the clonal growth assay. The observation that SINE compound treatment arrested both parental and resistant cells in G1 also correlated with gene expression data from the microarray results. The observation that a large fraction of resistant cells are arrested in G1 corresponded with a greater reduction in the expression of three genes important for G1-S transition, CCNE1, CCNE2, and CDC25A [reviewed in [61, 62]] and for S phase initiation, CDC6 [reviewed in [63]] in resistant compared to parental SINE compound treated cells.

The microarray results suggest that acquired resistance to SINE compounds is associated with combined modulation of adhesion-related genes, amplification of inflammation pathways, up-regulation of anti-apoptotic machinery coupled to down-regulation of pro-apoptotic pathways, as well as activation of immune evasion mechanisms. The comparison of the expression profile of adhesion pathway genes in untreated parental to resistant cells indicates that resistant cells have a more aggressive phenotype, which is typically characterized by increased invasion, metastatic ability, and resistance to therapy [64]. For example, the expression of HAS2, OPN, ITGB8, and MMP9 was higher in resistant cells. HAS2 (hyaluronin synthase 2) produces HA (hyaluronic acid) and its expression is significantly correlated with tumorigenicity and tumor progression in several cancers [65]; OPN (osteopontin) is a secretory adhesive protein overexpressed in a variety of cancers and its overexpression is correlated with poor prognosis [66]; ITGB8 (integrin beta 8) overexpression correlates with increased invasiveness [67]; and MMP9 (matrix metalloproteinase 9) enhances the invasion and metastasis of tumor cells [68] and its induction is a feature of activated fibroblasts, myofibroblasts [69] (also see changes in smooth muscle actin below). These changes in gene expression are also in agreement with phenotypic changes of resistant cells that were observed in culture, with increased adhesion to tissue culture dishes in comparison with parental cells (length of exposure to trypsin, not shown). In further support of changes in adhesion-related genes, significant down-regulation of NID2 (nidogen 2) was observed in resistant cells. Recent studies suggest that reduced expression of this gene correlates with higher rate of metastasis [70, 71] further supporting the theory that resistant cells have a more aggressive phenotype than parental cells.

The observation that resistant cells are more difficult to kill than parental cells, as evidenced by increased SINE compound IC_50_ values, persistent growth in the clonal assay, and less cleaved PARP and Caspase 3 proteins in resistant compared to parental cells, is further supported by changes in gene expression in the group of apoptosis related genes. Resistant cells induce the transcription of CLU (clusterin) and down-regulate the expression of PLAGL1 (pleiomorphic adenoma gene-like 1). CLU is overexpressed in several cancers and has been shown to inhibit apoptosis by interfering with Bax activation in mitochondria [72], while PLAGL1 is a tumor suppressor protein, which concurrently induces apoptosis and cell cycle arrest [73].

From the pro-inflammatory genes, Chemokine C-C motif ligand 2 (CCL2) was the most up-regulated in resistant versus parental cells, followed by another pro-inflammatory protein, TREM1 (triggering receptor expressed on myeloid cells 1). CCL2 has been implicated in promoting breast cancer metastasis [74] as well as prostate cancer growth [75] while TREM1 expression in hepatic satellite cells negatively correlated with disease outcome and its expression was related to aggressive tumor behavior [76]. The class II major histocompatibility complex determinant HLA-DPB1 was down-regulated in resistant cells. Failure to express Class I and/or Class II MHC determinants is a common feature of the majority of human prostatic carcinoma cells and may represent an immune evasion mechanism promoting tumor survival and metastasis [77].

Although expression changes for most genes were similar in parental and resistant cells in response to drug treatment, the extent of the change was generally stronger in parental cells. For example, NPY1R was upregulated 2.97 fold after drug treatment in parental cells, while only 1.77 fold in resistant cells. A few studies support an anti-proliferative and possibly pro-apoptotic role for NPY1R [78, 79], but this function requires activation by NPY and it is not clear whether a pure transcriptional event translates to an anti-proliferative function in-vitro. ID2 (inhibitor of DNA binding 2, dominant negative helix-loop-helix binding protein), which was found to be pro-apoptotic in osteosarcoma cells [80], was exclusively induced in parental cells. Several anti-apoptotic genes were exclusively down-regulated in parental cells. These include VEGFA, IL1B (interleukin 1 beta), and XBP1 (X-box binding protein 1). Inhibition of VEGFA production in tumor cells has previously been reported to induce apoptosis in-vitro [81] and a reduction in levels of VEGFA also suggests a potential anti-angiogenic effect of SINE compound treatment in vivo. Another notable difference is in the expression of EGR1 (early growth response 1), induced exclusively in resistant cells. EGR-1 may act as either a tumor promoter or suppressor, depending on the type of tumor [82]. In HT-1080 fibrosarcoma cells, EGR-1 was found to suppress cell growth by activating TGF-beta 1 [83] and in a recent study of synovial sarcoma, it was found to mediate cell death induced by HDAC inhibitor through activation of PTEN [84]. The up-regulation EGR-1 expression may thus represent an anti-proliferative mechanism unique to SINE compound activity in resistant HT-1080 cells.

Several changes were also observed in the proliferation-related response to drug treatment. As expected, a number of anti-proliferative genes were more strongly induced in parental cells, including BTG2 (BTG family, member 2), TOB1 (transducer of ERBB2, 1), SESN1 (sestrin 1), and GNG2 (guanine nucleotide binding protein). In contrast, EDN1 (endothelin 1), which is a known pro-survival protein in ovarian carcinoma [85], was more strongly induced in resistant cells, in line with a pro-survival response.

Finally, the transcriptional response of genes related to cell-cycle and cytoskeleton expression changes was largely similar in parental and resistant cells, with most of the genes mildly down-regulated. A few notable differences include p21, induced only in parental cells, which could be related to the higher expression of PLAGL1 in resistant cells [86], CCNE1 (cyclin E1), CCNE2 (cyclin E2) and CDC25A,which are exclusively down-regulated in resistant cells, HDAC9 which is up-regulated only in resistant cells, and ACTA2 (α smooth muscle actin), which is higher in resistant cells. HDAC9 was recently found to promote angiogenesis [87] and increased expression of ACTA2 is a hallmark of fibroblast transformation to myofibroblasts [69]. While the latter is also in accord with the increased expression of MMP9 by resistant HT1080 at baseline, ACTA2 is also a direct transcriptional target of the tumor suppressor p53 [88] so its role in the response to SINE compounds is unclear and remains to be explored. These patterns of changes in gene expression indicate that the response to SINE compound treatment described above is mostly pro-apoptotic, anti-proliferative, and cytostatic.

Validation of the microarray results provided many examples of genes that could be evaluated as potential biomarkers to predict response to treatment with SINE compounds. SLC16A6, SLC43A2, ARRDC3, NGFR, and HSPA4L were all up-regulated in response to drug treatment by all cell types tested including parental malignant, resistant malignant, and normal cell lines, as well as normal leukocytes isolated from healthy human blood. SLC16A6 functions as a proton-linked monocarboxylate transporter and was found to be significantly increased in drug resistant ovarian cancer cell lines [89]. SLC43A2 is a Na^+^-, Cl^−^-, and pH-independent high affinity transporter of large neutral amino acids whose role in cancer has not been determined [90]. Up-regulation of ARRDC3 would be a beneficial effect of SINE compound treatment as it was shown that its overexpression represses breast cancer cell proliferation and does so by negatively regulating beta-4 integrin [91]. Depending on the context, NGFR expression can either be oncogenic or tumor suppressive and recent studies with colon cancer indicate it has anti-tumor activity [92]. The role of the heat shock protein HSPA4L is unclear but the gene was found to have a hypermethylated promoter in leukemia cell lines [93]. Further studies are necessary to determine the functional relevance of the up-regulation of these genes in response to drug treatment.

## Conclusions

In summary, resistance to SINE compounds generated in HT1080 cells appears to be a reflection of reduced sensitivity of the overall system to XPO1 inhibition, and is not due to mutation of the target, prevention of drug binding, or drug efflux. Developed resistance is characterized by decreased potency of XPO1 inhibitors, altered cell cycle profile and less forced nuclear retention of XPO1 cargo. By evaluating global gene expression changes pre- and post-treatment, we have developed a profile of gene alterations relevant to SINE compound response and the development of resistance. Components of this profile include 1) genes that are altered when resistance is conferred in an originally SINE compound sensitive cell type, 2) genes whose expression is altered in parental cells in response to drug treatment, 3) genes whose expression is altered in resistant cells in response to drug treatment, and 4) a menu of genes whose expression is affected when XPO1 is inhibited in both malignant and normal cells. Both the large number of genes found, as well as the tendency for their expression to trend in the same direction (up or down) in both parental and resistant cells suggests that inhibiting XPO1 has a wide effect on downstream pathways and that this effect is more drastic in parental than resistant cells. Closer examination of the pathways identified will be necessary to provide a rationale for testing inhibition of specific targets in combination with SINE compounds to enhance the activity of SINE compound mono-therapy.

## Additional file

Additional file 1: Table S1. MetaCore analysis of fold changes in expression of all genes in SINE compound-resistant versus parental cells post-treatment. FDR = Benjamini-Hochberg False Discovery Rate. Red = positive, blue = negative, color intensity corresponds to fold change magnitude. **Table S2.** MetaCore analysis of fold changes in expression of genes with p53 regulatory elements in SINE compound-resistant versus parental cells post-treatment. FDR = Benjamini-Hochberg False Discovery Rate. In the p53 regulation column, “+”, “-“and “?” correspond to positive, negative, and uncertain regulation by p53, respectively. Red = positive, blue = negative, color intensity corresponds to fold change magnitude. (XLSX 104 kb)

## Abbreviations

SINE: Selective inhibitors of nuclear export
XPO1: Exportin 1
TSP: Tumor suppressor protein
FACS: Fluorescence activated cell sorting
qPCR: Quantitative polymerase chain reaction
NES: Nuclear export signal
LMB: Leptomycin B
